# Metabolic reprogramming and cisplatin resistance: the emerging role of Pyruvate Dehydrogenase Kinases (PDKs)

**DOI:** 10.1186/s12964-026-02845-9

**Published:** 2026-03-28

**Authors:** Aishath Shaheeda, Shama Prasada Kabekkodu

**Affiliations:** Department of Cell and Molecular Biology, Manipal School of Life Sciences, Manipal Academy of Higher Education, Manipal, India

**Keywords:** Pyruvate dehydrogenase kinase, Cisplatin resistance, Metabolic reprogramming, Warburg effect, Glycolysis, Redox homeostasis

## Abstract

Cisplatin resistance remains a primary obstacle in oncology, leading to therapeutic failure and disease recurrence. While resistance mechanisms are multifactorial, metabolic reprogramming has emerged as a critical adaptive strategy for cancer cells to evade cisplatin-induced cytotoxicity. This review synthesizes current evidence on how altered metabolism drives cisplatin resistance, with a particular emphasis on the pivotal role of pyruvate dehydrogenase kinases (PDKs). PDKs act as metabolic gatekeepers by inhibiting the pyruvate dehydrogenase complex (PDHC), diverting glucose flux away from mitochondrial oxidation toward glycolysis. This switch supports biomass production, maintains redox homeostasis, and dampens reactive oxygen species (ROS) generation, ultimately promoting cell survival. We detail how each PDK isoform (PDK1-4) contributes to resistance through distinct mechanisms, including the regulation of the DNA damage response, cancer stemness, and hypoxia signaling. Furthermore, we discuss the metabolic cues that regulate PDK activity and the role of tumor immune metabolism in cisplatin resistance. Furthermore, we discuss the metabolic cues that regulate PDK activity and the emerging role of tumor immunometabolism in the development of cisplatin resistance. Finally, we highlight the therapeutic potential of targeting PDKs, particularly through pharmacological inhibitors such as dichloroacetate (DCA), while also considering the current challenges associated with PDK-directed therapies. Overall, targeting PDK-mediated metabolic plasticity may offer a promising strategy to overcome cisplatin resistance and improve therapeutic outcomes.

## Introduction

Despite advances in diagnosis and treatment, cancer remains a leading cause of death worldwide. Chemotherapy is traditionally used for treating both solid and hematological malignancies and is used either as a standalone treatment or in combination with other therapeutic modalities [1–3]. Despite being a frontline treatment choice in early and metastatic cancers, chemotherapy is limited by its systemic toxicity and frequent development of resistance, which ultimately reduces its therapeutic index, compromises disease-free survival, and diminishes the quality of life among patients [4–6]. Cisplatin is the product of accidental discovery and quickly emerged as a primary treatment modality for a wide spectrum of solid tumors, both alone and in combination with surgery and radiotherapy [7].

**Fig. 1:**
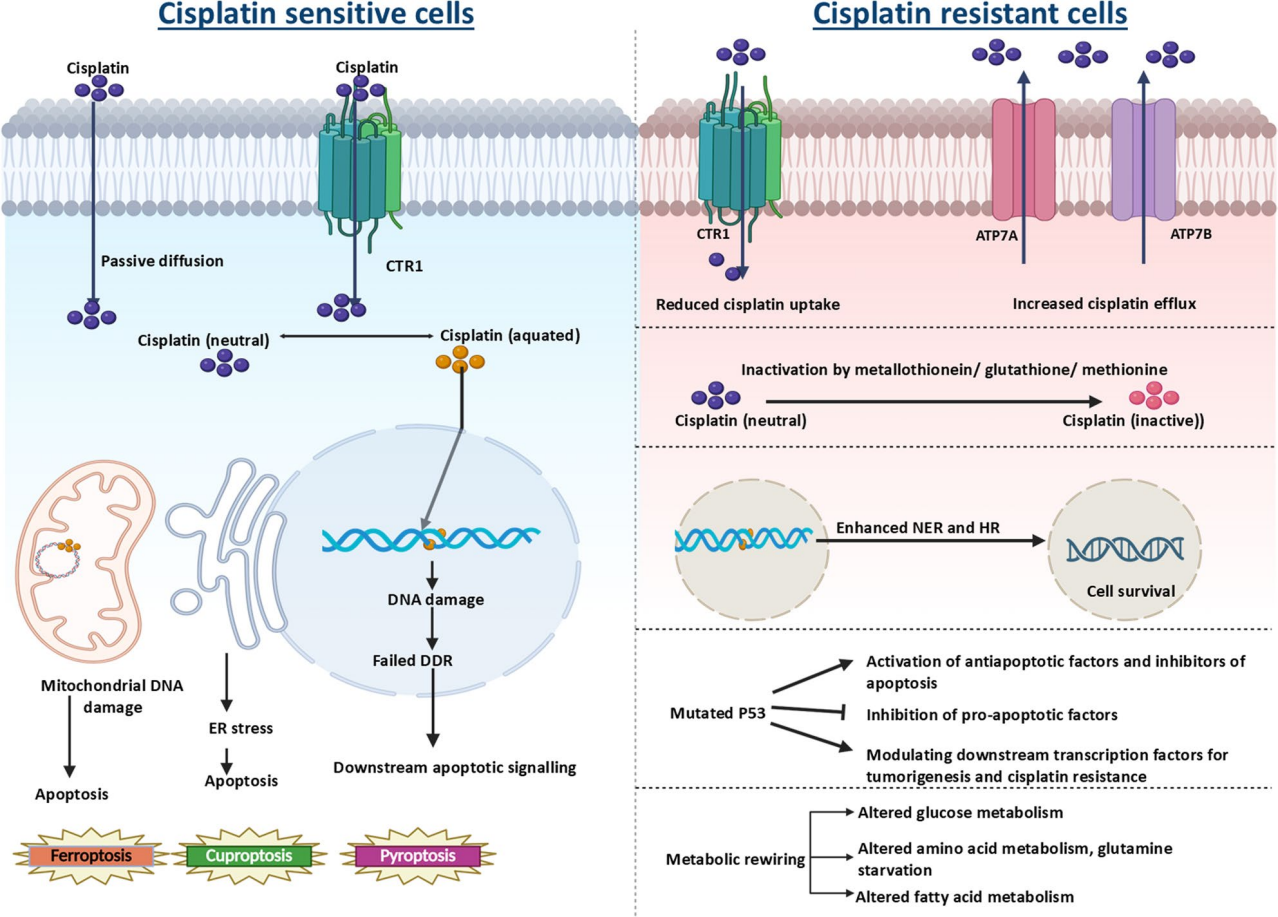
Mechanisms of action of cisplatin in sensitive cells and pathways contributing to resistance. Cisplatin causes cytotoxicity through DNA damage, ER stress, mtDNA damage and downstream apoptotic signaling, whereas resistant cells evade these effects through alterations in drug accumulation, inactivation by nucleophilic scavengers, DNA repair, cell death pathways and epigenetic and metabolic alterations. DDR (DNA damage repair), NER (nucleotide excision repair), HR (homologous recombination)

Cisplatin is a neutral square planar platinum complex consisting of a central platinum atom coordinated with two amine ligands, which stabilize binding, and two chloride ligands, which undergo aquation to enable covalent interaction with DNA [8]. The mechanism of action of cisplatin, as well as resistance mechanisms, has been comprehensively reviewed by Siddik et al. (2003) and Gu et al. (2025) [9, 10]. Upon entry via passive diffusion or active transport (Ctr 1), cisplatin is activated by aquation, which results in the formation of DNA adducts, hinders transcription, triggers DNA damage response (DDR) pathways, and culminates in cell death [11, 12]. In addition to causing nuclear and mitochondrial DNA damage, cisplatin causes endoplasmic reticulum (ER) stress and alters plasma membrane (PM) fluidity [13, 14]. Despite its efficacy, intrinsic and acquired drug resistance remain major challenges in long-term cancer treatment [15]. Mechanisms include reduced drug accumulation (Ctr 1 low, MDR and ATP7A/B high) [16, 17], enhanced DDR [18], detoxification by cytosolic nucleophiles [19], mutation of p53 [20], and impairment of apoptotic signaling [21]. Resistant cells often display inhibition of apoptosis as well as other forms of cell death, such as pyroptosis [22], ferroptosis [23], and cuproptosis [24]. Increasing evidence highlights that cisplatin resistance extends beyond genetic and epigenetic regulation, with metabolic reprogramming emerging as a central determinant of the therapeutic response. The contrasting mechanisms of action of cisplatin in sensitive versus resistant cells are illustrated in Fig. 1.

Tumor metabolism is a key driver of cisplatin resistance and orchestrates diverse metabolic pathways to support cancer cell survival. Dysregulated glucose, amino acid, and fatty acid metabolism are common features of resistant cells [25]. Enhanced aerobic glycolysis (the Warburg effect), in which glucose is converted to lactate despite oxygen availability, is strongly linked to cisplatin resistance [26]. In addition to glycolysis, oxidative phosphorylation (OXPHOS) [27], amino acid metabolism [28], and fatty acid metabolism [29] are altered in resistant cells to maintain redox homeostasis and buffer against cisplatin-induced oxidative stress. Tumor cells do not choose a metabolic pathway that is mutually exclusive to the other; rather, they represent adaptive programs that can be differentially engaged depending on treatment duration, tumor stage, and microenvironmental constraints to achieve better cellular fitness, including chemoresistance. PDKs function as central metabolic gatekeepers on this continuum by regulating pyruvate entry into the TCA cycle [30, 31].

Altered metabolic circuitry often compromises cisplatin cytotoxicity by facilitating nucleotide biosynthesis [32], sustaining stemness properties [33], maintaining ion homeostasis [33], reactivating embryonic genes [34], reshaping the tumor microenvironment [35], and suppressing apoptotic factor release and excessive ROS accumulation [36]. Collectively, this metabolic plasticity offers survival advantages that enable cells to evade drug-induced stress.

Among these factors, central carbon metabolism is profoundly important, as several enzymes and transporters of these pathways have been reported to be involved in cisplatin resistance. Pyruvate dehydrogenase kinases (PDKs) have emerged as particularly critical regulators because they occupy a central metabolic node to modulate metabolic circuitry by determining the balance between glycolysis and oxidative phosphorylation [37]. Through phosphorylation-mediated inhibition of the pyruvate dehydrogenase complex (PDHC), PDKs reduce pyruvate flux into the tricarboxylic acid (TCA) cycle, thereby shifting cellular metabolism toward glycolysis while attenuating mitochondrial oxidative activity and reactive oxygen species (ROS) generation [37]. As central regulatory nodes linking glycolysis and mitochondrial metabolism, PDKs act as critical metabolic gatekeepers that orchestrate metabolic plasticity and modulate cellular adaptation to cisplatin-induced stress, ultimately influencing the therapeutic response [37]. Several PDK inhibitors have exhibited potential therapeutic benefits both in vitro and in vivo [38]. Preclinical studies have reported that pharmacological inhibition of PDKs can restore cisplatin sensitivity in vitro and in vivo, underscoring their therapeutic potential [39]. Thus, PDKs represent a promising metabolic gateway for energy metabolism, redox balance, and therapeutic resistance. In this review, we provide a comprehensive overview of cellular metabolism in cisplatin resistance, with a particular focus on the role of PDKs as glycolytic gatekeepers and emerging therapeutic targets.

### Metabolic rewiring: a driver of cisplatin resistance

Tumorigenesis and drug resistance are highly dependent on metabolic reprogramming [25]. Cancer cells use metabolic rewiring as an efficient method to satisfy their energy needs, biosynthesis, and oxidation‒reduction reaction requirements to cope with the altered tumor microenvironment [40]. Cancer cells exhibit aberrant glycolysis, mitochondrial metabolism, amino acid metabolism, fatty acid metabolism, and redox metabolism, which favor cell survival under hostile conditions.

### Rewired carbon flux: a determinant of cisplatin sensitivity

Glucose is a principal carbon source; its catabolism through glycolysis and the tricarboxylic acid (TCA) cycle provides essential energy (ATP), biosynthetic precursors, and reducing equivalents (NADPH) necessary for cell survival and proliferation. In 1956, for the first time, Otto Warburg described the unusual reliance on glucose metabolism by cancer cells, termed the Warburg effect [26]. Cisplatin-resistant cells exhibit increased glucose uptake and overexpression of glycolysis-associated proteins [41, 42]. The inhibition of glycolysis has been demonstrated to increase cisplatin sensitivity [43]. Glucose transporter 1 (GLUT1) is frequently upregulated in cisplatin-resistant cells, and pharmacological inhibition with WZB11, a small-molecule GLUT1 inhibitor, synergistically sensitized human nontumorigenic NL20 lung and MCF12A breast epithelial cells [44], head and neck squamous cell carcinoma (HNSCC) cells [45], esophageal cancer cells [46], and oral cancer cells to cisplatin [47].

Hexokinase 2 (HK2), the enzyme that catalyzes the first rate-limiting step of glycolysis, is often overexpressed in cancers [48]. In bladder cancer, HK 2 is positively regulated by the tyrosine kinase SRC, which enhances glycolysis and the pentose phosphate pathway, ultimately conferring cisplatin resistance. Silencing SRC, thereby suppressing HK2 activity, efficiently reversed cisplatin resistance in bladder cancer [41]. Similarly, targeting HK2 with miR-143 enhances cisplatin sensitivity by inhibiting glycolysis [49]. In addition to their metabolic role, HK isoforms can bind to voltage-dependent anion channels (VDACs) on the outer mitochondrial membrane, reducing channel conductance, blocking cytochrome c release, and protecting against cisplatin-induced apoptosis [50].

Another glycolytic regulator, 6-phosphofructo-2-kinase/fructose-2,6-biphosphatase 3 (PFKFB3), which is uniquely localized in the nucleus, has also been implicated in cisplatin resistance. Li et al. (2018) reported that cisplatin induces the acetylation of PFKFB3 at lysine 472, driving its localization from the nucleus to the cytosol, where it promotes glycolytic flux and protects against cisplatin cytotoxicity via AMPK-mediated phosphorylation. They also reported that the use of a potent inhibitor of PFKB3, such as PFK15, potentiated cisplatin cytotoxicity and suppressed tumor growth in xenograft models [51]. PFKB3 blockade promoted vascular normalization and improved cisplatin sensitivity in endothelial cancer [52].

Another glycolytic player, enolase 1 (ENO1), is upregulated in gastric cancer, and its depletion reversed cisplatin resistance [53].

Pyruvate kinase M (PKM), a critical regulator of glycolysis, exists in two isoforms, PKM1 (adult) and PKM2 (embryonic). Tumor cells preferentially express PKM2, which supports malignancy and resistance. Silencing PKM2 and substituting it with PKM1 reversed the Warburg effect in xenografts [54]. PKM2 is overexpressed in metastatic nonsquamous cell carcinoma [55] and glioblastoma [34] and is correlated with the response to cisplatin and overall survival. In squamous cell carcinoma, PKM2 silencing enhances cisplatin sensitivity through autophagy regulation [56]. In bladder cancer, cisplatin-resistant cells overexpress PKM2, and its depletion restores cisplatin cytotoxicity [57]. Moreover, resistant cells can transfer PKM2 via exosomes under hypoxia, conferring resistance to sensitive tumor cells [58]. In addition to its metabolic functions, PKM2 plays noncanonical roles in gene transcription, epigenetic regulation, and cell signaling, which are crucial in cancer metastasis. In pancreatic cancer, cancer-associated fibroblasts (CAFs) drive resistance to platinum through the nuclear translocation of PKM2, the upregulation of the expression of glycolysis-related genes, and the secretion of IL-8, all of which enhance DNA damage repair [59].

Lactate dehydrogenase (LDH), which is responsible for converting pyruvate to lactate, also contributes to resistance. Elevated lactate promotes the expression of DNA repair genes, facilitating cisplatin resistance [60]. LDH inhibitors such as oxamate and galloflavin are used to sensitize Burkitt lymphoma cells to cisplatin by increasing ROS generation [61]. Silencing LDHB impaired nucleotide biosynthesis and promoted nuclear DNA damage [62]. Compounds such as alantolactone, which target glycolytic enzymes, including LDHA, PFKL, and HK2, have also been shown to increase cisplatin sensitivity in ovarian cancer [63]. The pyruvate dehydrogenase complex (PDHC) is a critical gatekeeper that converts pyruvate to acetyl-CoA and is regulated by pyruvate dehydrogenase kinases (PDKs) and phosphatases, of which PDKs are increasingly recognized and are discussed in detail in the following sections.

While Warburg originally proposed that tumor cells rely predominantly on glycolysis because of defective mitochondrial respiration, accumulating evidence indicates that oxidative phosphorylation (OXPHOS) remains functional in many cancers and can contribute to cisplatin resistance [64]. The choice between glycolysis and OXPHOS in cancer cells is not mutually exclusive; rather, these metabolic states exist along a continuum that enables adaptive survival under cisplatin-induced stress. Enhanced glycolysis is frequently observed as an early acute response to cisplatin exposure, supporting DNA repair and antioxidant production [41]. However, with prolonged treatment, cancer cells can adapt by increasing their reliance on mitochondrial respiration and OXPHOS [65, 66]. In hypoxic tumors, the stabilization of hypoxia-inducible factor-1α (HIF-1α) transcriptionally upregulates the expression of glycolytic enzymes, including PDKs, thereby limiting excessive oxidative stress and promoting resistance [67]. Conversely, cancer stem cells (CSCs) preferentially rely on OXPHOS to meet the bioenergetic demands required for maintaining stemness, thereby contributing to therapeutic resistance and tumor recurrence [68]. Thus, the apparent glycolysis–OXPHOS shift is shaped by multiple factors, including tumor stage, hypoxic stress, oncogenic signaling context, and duration of therapeutic exposure [69, 70].

In contrast, cancer stem cells (CSCs) often exhibit increased dependence on OXPHOS to sustain bioenergetic demands associated with stemness and tumor recurrence [70]. Thus, the apparent glycolysis–OXPHOS shift is shaped by multiple factors, including tumor stage, hypoxic stress, oncogenic signaling context, and duration of therapeutic exposure [71, 72].

HIF-1α is a well-studied transcription factor that regulates glycolytic enzymes. Cisplatin treatment induces the degradation of HIF-1α in sensitive cells, redirecting glycolysis toward mitochondrial respiration and ROS generation, thereby promoting apoptosis. In resistant cells, however, HIF-1α is stabilized, and its knockdown resensitizes cells to cisplatin [71]. The overexpression of LDHA counteracts HIF-1α degradation, reducing sensitivity [71]. Increased sensitivity to OXPHOS-associated cisplatin has been reported in high-grade serous ovarian cancer via regulation of the promyelocytic leukemia protein-peroxisome proliferator-activated receptor gamma coactivator-1α (PML-PGC-1α) axis [27]. Conversely, fluorescence lifetime microscopy (FLIM) revealed that cisplatin treatment upregulated mitochondrial genes, promoted oxidative phosphorylation and contributed to resistance; moreover, the inhibition of OXPHOS genes reversed the FLIM metabolic signature and decreased cisplatin resistance [72].

OXPHOS inhibitors combined with cisplatin increase cisplatin sensitivity in an aldehyde dehydrogenase (ALDH)-dependent manner [73]. Cisplatin induces early cytosolic acidification, and resistant cells adapt by switching to OXPHOS to buffer this environment, reducing drug efficacy [74]. OXPHOS increases stemness in colorectal cancer, and its inhibition by sponging PGC1-α sensitizes cells to cisplatin [68]. Mutations in mitochondrial transcription factor A (TFAM) confer resistance by regulating cytochrome b transcription and release.

OXPHOS enzymes have also been reported to play a role in cisplatin resistance. Citrate synthase (CS) silencing resulted in increased cisplatin sensitivity in ovarian cancer. Citrate supplementation markedly reduces cancer progression and enhances cisplatin sensitivity [75]. In addition, sodium citrate supplementation induces apoptosis and ferroptosis through excessive ROS [76]. The upregulation of isocitrate dehydrogenase (IDH) [77], aconitase [78], and α-ketoglutarate dehydrogenase (OGDH) [79] modulates ROS levels and cisplatin sensitivity, whereas succinate dehydrogenase (SDH) deficiency stabilizes HIF-1α activity and drives glycolysis-dependent resistance [80, 81]. Loss of fumarate hydratase (FH) promotes fumarate accumulation, inhibition of mitochondrial replication factors and thus mtDNA mutation, culminating in glycolytic dependency and drug resistance [82]. Similarly, cisplatin-resistant ovarian cancer cells exhibit increased expression of CS, IDH, and malate dehydrogenase (MDH) [63].

The pentose phosphate pathway (PPP) also contributes to cisplatin resistance by generating NADPH for redox balance and lipid biosynthesis and by supplying nucleotides for DNA repair. The role of the PPP in cisplatin resistance was reviewed in detail by Giacomini et al. (2020) [32]. Nicotinamide adenine dinucleotide phosphate (NADPH), a primary byproduct of the PPP, is crucial for redox metabolism and lipid biosynthesis. PPP is often overexpressed in cisplatin-resistant cells [41]. In cisplatin-resistant cells, the expression of PPP enzymes such as glucose-6-phosphate dehydrogenase (G6PDH) is upregulated, and its silencing reverses resistance [83]. Ras-related C3 botulinum toxin substrate 1 (Rac1)-mediated overexpression of the aldolase enzyme results in the overexpression of the nonoxidative branch of the PPP and confers cisplatin resistance via increased nucleotide synthesis and DNA repair. Codelivery of Rac1 siRNA effectively resensitized cells to cisplatin [84]. Similarly, transketolase attenuates cisplatin sensitivity through GSH depletion and ROS accumulation in glioblastoma [85].

Pharmacological inhibitors of carbon metabolism have emerged as promising therapeutic strategies. Several glycolytic inhibitors, including BAY-876,2-deoxyglucose (2-DG), 3-bromopyruvate (3-BrPA), and plant-derived compounds such as genistein, methyl jasmonate, and shikonin, are under preclinical and early clinical evaluation. Bay-876 not only has antiproliferative effects but also significantly hinders epithelial‒mesenchymal transition (EMT) in hepatocellular carcinoma (HCC) [86]. 2-DG exhibited clinical potential at 63 mg/kg in combination with docetaxel and is advancing to phase II trials, although hypoglycemia associated with tolerable side effects remains a limitation [87]. AZD3965, an MCT1 inhibitor, has shown encouraging results with manageable ocular side effects and is recommended for tumors with high MCT1 and low MCT4 expression [88]. Additionally, 3-BrPA, a small-molecule glycolytic inhibitor that is selectively taken up through monocarboxylate transporter 1 (MCT1), has been shown to have strong antitumor effects in murine models by disrupting HK2-VDAC interactions and lowering mitochondrial hyperpolarization [89]. However, its alkylating activity causes significant cytotoxicity, limiting its clinical application [90]. Less toxic derivatives, such as KAT-101 and KAT-201, are now being evaluated in HCC [91]. Genistein, a phytoestrogen and HIF-1α inhibitor, has shown promise in the treatment of colorectal cancer when combined with bevacizumab [92]. Similarly, methyl jasmonate and shikonin exhibit antitumor effects in vitro and in vivo, but their toxicity limits their clinical translation [93, 94].

In addition to glycolysis, inhibitors targeting lactate dehydrogenase and oxidative phosphorylation are being explored. (R)-GNE-140, an LDHA/B inhibitor, and BMS-986,205, an IDO1 inhibitor, have demonstrated antiproliferative effects in tumor cells. Notably, BMS-986,205 has also been reported to exert off-target inhibitory effects on mitochondrial complex I, thereby impairing oxidative phosphorylation and ATP production. The combined inhibition of glycolysis and OXPHOS induces energetic catastrophe, resulting in reduced tumor cell proliferation [95]. Additionally, BMS-986,205 exhibited a manageable safety profile with durable benefits in an HCC cohort in a phase I/II trial [96]. In addition, necrocide-1 (NC1), another LDH inhibitor, exhibits promising antitumor effects through the suppression of glycolysis in vitro [97]. Collectively, these findings suggest that targeting central carbon metabolism represents a promising therapeutic strategy; however, the selection of metabolic inhibitors should be guided by the dominant metabolic phenotype of resistant cancer cells.

### Metabolic flexibility through amino acid rewiring

Cisplatin-resistant cells often move beyond the Warburg effect and exploit alternative metabolic pathways, such as lactate uptake and lipid and amino acid metabolism, to sustain survival and fitness [65]. Notably, many aggressive cancers are not affected by glucose deprivation and do not rely exclusively on conventional glycolysis for energy. Instead, they rewire their metabolism toward OXPHOS, which heavily depends on amino acid metabolism [98]. Amino acid metabolism drives cisplatin resistance by maintaining energy generation, redox homeostasis, and biomass synthesis, largely through the regulation of transporters that control intercellular and subcellular amino acid pools [28].

Glutamine is the most extensively studied amino acid in this context. Resistant cells exhibit glutamine addiction for survival, and their antioxidant capacity is tightly linked to glutamine availability and catabolism [99]. It functions as a pleotropic carbon and nitrogen donor for nucleotide biosynthesis, fuelling anaplerotic reactions that replenish α-ketoglutarate in the TCA cycle and serve as a precursor for glutathione (GSH) biosynthesis [28]. In resistant cells, glutamine synthase (GS) is downregulated through promoter methylation, whereas glutaminase (GLS) remains active. This imbalance promotes glutamine starvation, enhances glutaminolysis, and elevates GSH levels, thereby supporting redox homeostasis [100]. A recent study revealed that silencing glutamic pyruvate transaminase 2 (GPT2) reduces glutaminolysis and impairs TCA cycle replenishment by hindering the anaplerosis of glutamine, ultimately resensitizing resistant cells to cisplatin [101].

Serine metabolism is another important contributor to cisplatin resistance. Although serine synthesis promotes oncogenesis [102], paradoxically, the inhibition of serine biosynthesis has been reported to drive cisplatin resistance [103]. Similarly, tryptophan (Trp) catabolism via the kynurenine (KYN) pathway (KP) has emerged as an important mechanism of resistance. Cisplatin-resistant cells prefer KPs to overcome excessive ROS and maintain redox homeostasis. Clinically, patients with a poor cisplatin response have an elevated serum KYN/Trp ratio [104]. Moreover, indoleamine 2,3-dioxygenase-1 (IDO1), the rate-limiting enzyme of KP, is overexpressed in resistant tumors, and its inhibition restores cisplatin sensitivity [105].

Methionine metabolism further contributes to resistance by promoting cancer stemness. The inhibition of methionine adenosine transferase 2 A (MAT2A), which catalyzes the conversion of methionine to S-adenosylmethionine (SAM), reduces methionine flux, alters the tumor microenvironment, and synergistically enhances the efficacy of cisplatin [35].

The inhibition of amino acid uptake by targeting L-type amino acid transporter 1 (LAT1) has been shown to result in a positive chemotherapeutic response. JPH203, a selective LAT1 inhibitor, demonstrated favorable outcomes at 12 mg/m² and 25 mg/m² in patients with advanced solid tumors, with 25 mg/m² recommended for phase II trials [106]. Glutamine transporters (ASCT1/2) are inhibited by compounds such as V-9302 [107] and C118P [108], which induce cytotoxicity in breast cancer models. In parallel, glutaminase (GLS) inhibitors, including CB-839 (Telaglenastat), IACS-6274, and 6-diazo-5-oxo-L-norleucine (DON), are under clinical evaluation. CB-839 exhibited promising outcomes in metastatic melanoma, renal cell carcinoma, and non-small cell lung cancer (NSCLC) [109] and is currently in phase I trials for hematological malignancies (NCT02071888). IACS-6274 has positive outcomes in molecularly selected solid tumors [110] and is being tested in combination with bevacizumab and paclitaxel (NCT05039801). DON showed antitumor activity in early trials, but further progress has been hindered by toxicity [111]. Sulfasalazine (SASP), an FDA-approved anti-inflammatory drug, inhibits the xCT (cystine transporter) transporter and has antiproliferative effects on NSCLC [112].

Taken together, these findings suggest that targeting amino acid metabolism represents a promising therapeutic avenue. Strategic combinations of drug treatment with dietary or metabolic interventions could provide powerful approaches to overcome cisplatin resistance.

### Fatty acid metabolism to buffer against cisplatin stress

Fatty acid metabolism is an integral component of cancer cell physiology and plays pleiotropic roles, ranging from membrane biosynthesis and signaling intermediates to biomass production, energy generation, and posttranslational modification [113]. Cisplatin-resistant cells exhibit increased fatty acid uptake and favor beta-oxidation to survive under cisplatin-induced oxidative stress [29]. Hence, it is not surprising that resistant cells exhibit increased expression of these enzymes as well as precursors involved in fatty acid synthesis. Under nutrient-deprived conditions, exogenously acquired acetate is converted into acetyl-CoA by acetyl-CoA synthetases (ACSSs), whose expression is upregulated in cisplatin-resistant breast cancer cells [114]. Fatty acid synthase (FASN), a rate-limiting enzyme in de novo fatty acid synthesis, is similarly elevated in resistant cells, promoting the production of monounsaturated fatty acids that undergo less lipid peroxidation, thereby conferring greater resistance [23].

Several FASN inhibitors, such as Fasnall, GSK2194069, IPI-9119, and orlistat, have demonstrated potent antitumor effects; however, the associated adverse effects halt their clinical translation [115]. TVB-2640 is a FASN inhibitor that has entered a phase II clinical trial for astrocytoma treatment and has a tolerable drug and positive antitumor response along with bevacizumab, with an overall response rate (ORR) of 56% [116]. Moreover, it is currently under clinical trial as a combination therapy with paclitaxel for the treatment of HER2-positive metastatic breast cancer (NCT03179904). MTB-9655, an ACSS2 inhibitor, exhibited tolerable positive outcomes in preclinical studies using rodent and nonhuman primate models [117]. Other pharmacokinetic inhibitors, such as ND-646, TOFA, and GSK2194069, inhibit acetyl-CoA carboxylase 1 (ACC1) and FASN subunits but have no significant translational benefits because of their cytotoxicity [115, 118].

**Fig. 2:**
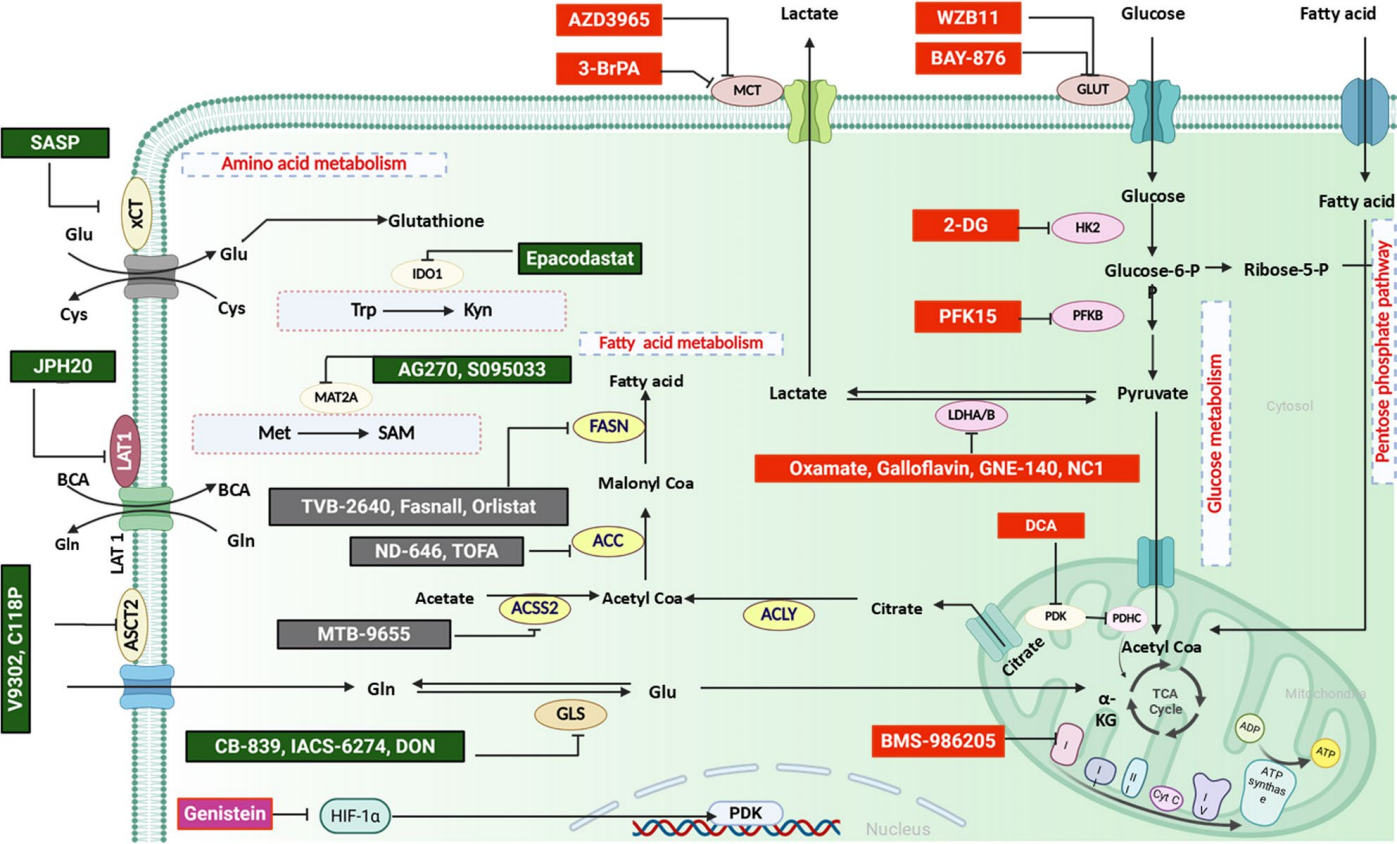
Schematic diagram of pharmacological inhibitors targeting different metabolic pathways. Inhibitors of glucose metabolism are shown in red, those of amino acid metabolism are shown in green, and those of fatty acid metabolism are shown in gray. 3-BrPA (3-bromopyruvate), 2-DG (2-deoxyglucose), Nec-1 (necrocide-1), DCA (dichloroacetate), DON (6-diazo-5-oxo-L-norleucine), and SASP (sulfasalazine)

Collectively, cancer cells exploit diverse metabolic alterations to evade the stress caused by cisplatin, resulting in cisplatin resistance. A schematic overview of pharmacological inhibitors targeting distinct metabolic pathways is illustrated in Fig. 2. Additionally, distinct metabolic regulatory factors and their key players in cisplatin resistance are summarized in Table 1.

**Table 1:** Metabolic plasticity in cisplatin resistance

Metabolic pathway	Key enzymes /transporters	Expression in -resistant cells	Therapeutic target	Reference
Glucose metabolism	GLUT1 HK2 PFKFB3 ENO1 PKM2 LDHA MCT PDK	High	• WZB11, BAY-876 (GLUT1 inhibitor) • 2-DG (HK2 inhibitor) • PFK15 (PFKFB3 inhibitor) • AZD3965, 3-BrPA (MCT1 inhibitor) • Oxamate, galloflavin, (R)-GNE-140, NC1 (LDH inhibitors) • Alantolactone targeting (HK2/LDHA) • DCA (PDK inhibitor)	[47, 50, 52, 55, 61, 81–88, 90, 95–97, 119, 120]
Pentose phosphate pathway	G6PDH ALDO	High	• siRNA-mediated silencing of RAC activator	[42, 121]
Mitochondrial metabolism	HIF-1α CS ACO IDH MDH TFAM OGDH SDH FH	High	• Genistein (HIF-1α inhibitor) • Citrate supplementation • BMS-986205(Complex 1 inhibitor)	[75, 77, 78, 80, 87, 92, 122, 123]
Amino acid metabolism	GS LAT xCT ASCT1/2 GLS GPT2 IDO1 MAT2A	Low	• Dietary modulation of glutamine and methionine	[100, 102, 104, 106–112, 123]
		High	• JPH20 (LAT inhibitor) • V9302, C118P, (ASCT1/2 inhibitors) • CB-839, IACS-6274, DON (GLS inhibitors) • SASP (xCT inhibitor) • AG-270/S095033 (MAT2A inhibitor) • Epacadostat (IDO1 inhibitor)	
Fatty acid metabolism	FASN ACSS ACC1	High	• siRNA-mediated silencing of ACSS/FASN • Fasnall, GSK2194069, IPI-9119, orlistat, TVB-2640 (FASN inhibitors) • MTB-9655 (ACSS inhibitor) • ND-646, TOFA	[115–118]

### Pyruvate dehydrogenase kinases (PDKs): The metabolic checkpoint between glycolysis and the TCA cycle

Previously, we discussed the diverse metabolic adaptations that cancer cells employ to survive under stress. Among these, pyruvate dehydrogenase kinases (PDKs) have emerged as central regulators at the intersection of multiple resistance-associated pathways. PDKs promote a metabolic shift toward the Warburg phenotype and contribute to cisplatin resistance by regulating the activity of the pyruvate dehydrogenase complex (PDHC), thereby modulating the magnitude and direction of metabolic flux in cancer cells in a context-dependent manner [37]. By phosphorylating and inhibiting PDHC, PDKs function as key metabolic gatekeepers that restrict the entry of glycolysis-derived pyruvate into mitochondrial oxidative metabolism. The PDHC consists of three enzymatic subunits: pyruvate decarboxylase (E1), dihydrolipoamide acetyl transferase (E2), and dihydrolipoamide dehydrogenase (E3). In addition, eukaryotic complexes contain a structural E3-binding protein (E3BP, or protein X), which lacks enzymatic activity. The activity of PDHC is governed primarily by its phosphorylation state, which is mediated by tightly associated kinases (PDKs) and more loosely associated phosphatases (PDPs), which together determine the flux of acetyl-CoA into the tricarboxylic acid (TCA) cycle [119]. The inhibition of PDHC activity conserves pyruvate for alternative fates, including NAD⁺ regeneration via lactate dehydrogenase, anaplerotic replenishment of oxaloacetate by pyruvate carboxylase, and transamination through alanine aminotransferase. Collectively, these pathways support cell proliferation, redox balance, and cisplatin resistance [120, 121].

PDKs, which are localized in the mitochondria, are composed of four isoforms (PDK1–4), are expressed in a tissue-specific manner and share approximately 70% sequence homology [37]. PDKs inactivate PDHC activity by phosphorylating serine residues Ser293 (site 1), Ser300 (site 2), and Ser232 (site 3) of the E1α subunit, a process facilitated by their interaction with the L2 domain of the E2 subunit [122].

### PDK1

PDK1 is expressed predominantly in the heart, pancreatic islets, and skeletal muscle. It is phosphorylated at three tyrosine residues (Tyr136, Tyr243, and Tyr244) by oncogenic kinases such as FGFR1 and BCR-ABL, which enhances ATP binding at its active site and promotes its association with the PDHC [123]. PDK1 overexpression is frequently associated with tumorigenesis [124]. Under hypoxia, HIF-1α induces PDK1 expression, thereby attenuating mitochondrial ROS production and sustaining ATP generation [125].

PDK1 is upregulated in hepatocellular carcinoma (HCC) via the circARNT2/miR-155-5p axis and confers cisplatin resistance, whereas circARNT2 knockdown restores drug sensitivity in HCC cells [126]. Similarly, in osteosarcoma, elevated PDK1 expression promotes metabolic reprogramming toward the Warburg phenotype, enhancing tumorigenicity and stemness through the activity of the ATF3/TGF-β/SMAD pathway and ultimately driving cisplatin resistance [127]. In ovarian carcinoma, high PDK1 levels are correlated with metastasis, poor drug sensitivity, and poor survival outcomes through the regulation of α5β1 integrin and JNK/IL-8 signaling. Notably, cancer-associated fibroblast (CAF)-derived IL-8 drives PDK1 upregulation in resistant cells [128]. PDK1 also upregulates epidermal growth factor receptor (EGFR) phosphorylation in cisplatin-resistant ovarian cancer, further increasing resistance, silencing PDK1 or pharmacologically inhibiting EGFR kinase activity (e.g., erlotinib) to significantly restore cisplatin sensitivity [129]. RNAi-mediated attenuation of PDK1 and EGFR expression reduces HIF-1α expression and shifts the Warburg phenotype to OXPHOS in glioblastoma [36]. Additionally, PDK1 expression is transcriptionally regulated by aryl hydrocarbon receptor nuclear translocator (ARNT) via NRF2. ARNT deficiency downregulates PDK1 expression and sensitizes cells to cisplatin through increased mitochondrial ROS levels, DNA fragmentation, and caspase 3 activation [130, 131]. Hypoxia has also been shown to drive the accumulation of mitochondrial Akt, which phosphorylates PDK1 at Thr346, inactivates PDHC, and reinforces the Warburg phenotype, a hallmark of cisplatin resistance [132].

**Fig. 3:**
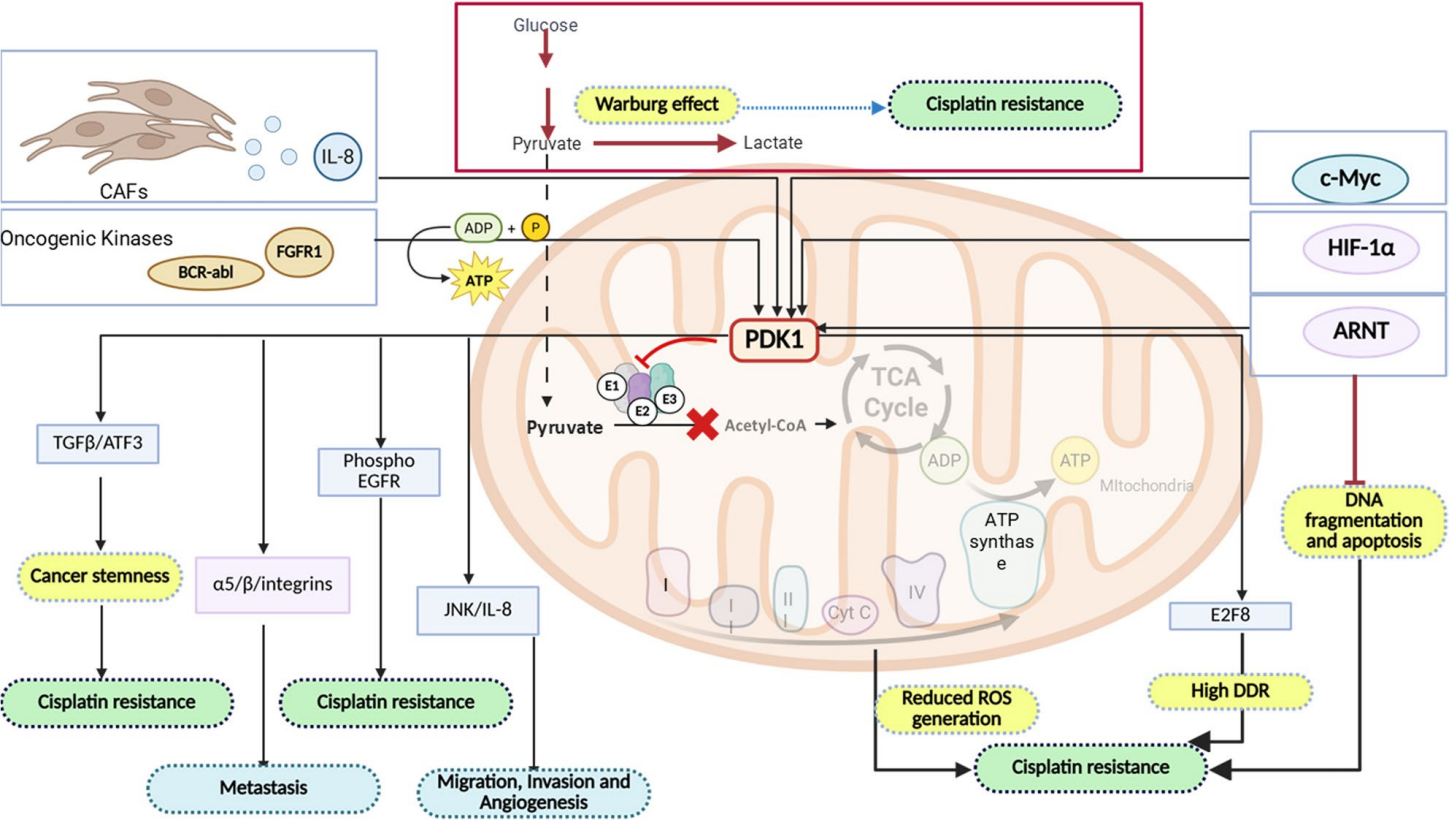
Schematic representation of PDK1-induced cisplatin resistance in cancer cells through the regulation of various transcription factors

Reduced expression of PGC-1α, a mitochondrial biogenesis factor, has previously been implicated in cisplatin resistance through HSP70-mediated mitochondrial accumulation of HK2, its interaction with VDAC, and the inhibition of cytochrome c release via altered mitochondrial membrane potential [50, 133]. In lung adenocarcinoma (LUAD), PDK1 is markedly upregulated and promotes cisplatin resistance by enhancing the DNA damage response through the E2F8/PDK1 axis [134]. Interestingly, recent work has shown that HIF-1α–driven activation of PDK1 and PDK3 contributes to cuproptosis resistance by reducing the expression of the PDHC component DLAT (a copper target) and increasing that of metallothionein-2 A (MT2A), which sequesters mitochondrial copper [24] (Fig. 3).

### PDK2

PDK2 is widely distributed in the spleen and lungs [37]. It has the highest binding specificity for site 1 of the E1 subunit in PDHC [37]. It is the only PDK isoform reported as a downstream transcriptional target of p53 [135]. Like PDK1, PDK2 also has oncogenic features, with higher expression levels correlated with poor prognosis and cisplatin resistance. The inhibition of PDK2 synergistically enhances the efficacy of cisplatin by stimulating the electron transport chain and increasing mitochondrial ROS production [136].

Mechanistically, PDK2 rewires cellular metabolism toward glycolysis, facilitating HK2 translocation to the outer mitochondrial membrane and inducing mitochondrial hyperpolarization, ultimately leading to cisplatin resistance. Treatment with the PDK2 inhibitor dichloroacetate (DCA) restores cisplatin sensitivity by promoting glucose oxidation, facilitating pyruvate entry into mitochondria, and reducing the mitochondrial membrane potential [137]. PDK2 is among the most upregulated kinases in cisplatin-resistant LUAD cells. It induces cisplatin resistance via the transcriptional upregulation of Cyclin M-3 (CNNM3), which regulates ion homeostasis and stemness-associated phenotypes, promoting resistance [33].

At the transcriptional level, PDK2 is negatively regulated by wild-type p53, which suppresses E2F activity through the p53/p21/E2F axis. Since E2F is a transcriptional activator of PDK2, its inhibition prevents Warburg metabolism, reduces lactate production, and promotes pyruvate flux into acetyl-CoA, thereby attenuating cisplatin resistance [135]. Conversely, mitochondrial ND2 mutations increase ROS accumulation, stabilize HIF-1α, and increase PDK2 activity, further suppressing PDHC and reinforcing resistance [138]. PDK2 reportedly augments cancer stemness by modulating the expression of transforming growth factor-beta (TGFβ) and promoting cisplatin resistance in HNSCC tissues. Silencing both PDK1 and PDK2 suppresses TGF-β activity, reduces stemness, and restores cisplatin sensitivity [139].

**Fig. 4:**
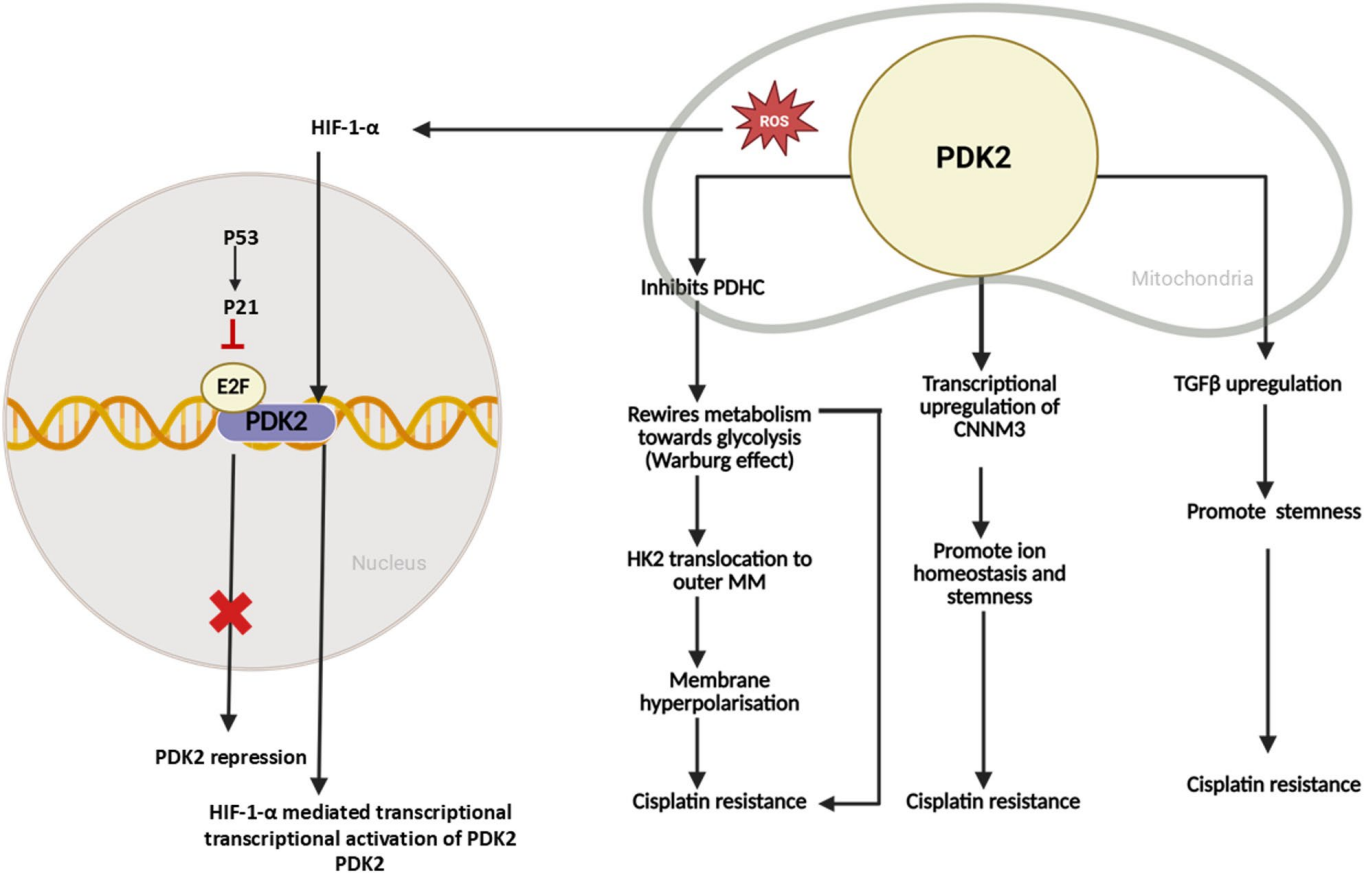
Schematic representation of how PDK2 causes cisplatin resistance in cancer through the regulation of various pathways

Furthermore, inhibition of PDK2 synergistically increased cisplatin sensitivity by activating the electron transport chain and increasing the production of mitochondrial ROS. In mouse xenograft models, inhibition of PDK2 in combination with cisplatin treatment significantly impaired tumor growth. This evidence suggests that targeting mitochondrial metabolism and redox homeostasis is an attractive therapeutic strategy for improving drug sensitivity in ovarian cancer. PDK2 regulates cisplatin resistance predominantly by abrogating the Warburg effect and ROS production, promoting cancer stemness, regulating ion homeostasis and altering the mitochondrial membrane potential. In summary, PDK2 contributes to cisplatin resistance by sustaining glycolytic metabolism, altering the mitochondrial membrane potential, regulating ion homeostasis, enhancing stemness, and modulating ROS signaling. (Fig. 4)

### PDK3

PDK3 expression is limited to the testicle, kidney, and brain [37]. Among the four isoforms, PDK3 has the strongest binding affinity for the E2 domain of PDHC [122]. It has the highest kinase activity and the least sensitivity to negative feedback inhibition by the pyruvate concentration [140]. Elevated PDK3 expression has been reported in multiple cancers and is positively correlated with cisplatin resistance. In colon cancer, high PDK3 levels promote resistance through the HIF-1α/PDK3 axis [67]. Mechanistically, PDK3 upregulation abrogates cisplatin sensitivity by promoting lactate accumulation, enhancing aerobic glycolysis, and suppressing mitochondrial respiration [141].

Interestingly, PDK3 is transcriptionally regulated by heat shock factor 1 (HSF1), and the expression of both proteins is reinforced through a positive feedback loop. Nucleus-localized PDK3 can inhibit the GSK3β-mediated phosphorylation of HSF1, thereby preventing FBXW7-dependent polyubiquitination and proteasomal degradation of phosphorylated HSF1 [142]. In addition, recent evidence has demonstrated that the PDK1/PDK3 metabolic switch is transcriptionally regulated by E2F1, which is coactivated by the histone lysine demethylase KDM4A. This KDM4A–E2F1–PDK3 axis rescues cisplatin cytotoxicity by favoring glycolysis over mitochondrial oxidation, while suppression of either KDM4A or PDK3 expression restores cisplatin sensitivity [143].

At the posttranscriptional level, PDK3 expression is negatively regulated by miR-497-5p in gastric cancer [144]. Similarly, reactivation of the chromosome 14 miRNA cluster (C14MC) targets PDK3 and suppresses its tumorigenic properties in cervical cancer cells [145]. Collectively, these findings highlight PDK3 as a critical mediator of metabolic plasticity and cisplatin resistance.

### PDK4

PDK4 is expressed primarily in muscle tissue and the liver, but it is also expressed in epithelial cells such as the bladder, where it is frequently dysregulated during cancer progression [120]. In bladder cancer, compared with that in low-grade tumors, PDK4 expression is elevated up to 33-fold in high-grade invasive tumors. Silencing PDK4 with siRNA reversed cancer phenotypes, and combined treatment with cisplatin and the PDK inhibitor dichloroacetate significantly enhanced therapeutic efficacy by inducing intratumoral necrosis [146]. They also reported that baseline upregulation of HIF-1α and peroxisome proliferator-activated receptor-α (PPARα) in these cells may contribute to the transcriptional activation of PDK4.

Mechanistically, PDK4 upregulation enhances the transcription of endothelial PAS domain-containing protein 1 (EPAS1), a regulator of oxidative stress resistance, angiogenesis, and glucose metabolism, thereby promoting cisplatin resistance [147]. Conversely, other studies reported a tumor-suppressive role for PDK4, showing that it inhibits TGFβ-induced epithelial–mesenchymal transition (EMT) and that its silencing confers erlotinib resistance. In addition, reduced PDK4 expression has been reported to predict poor prognosis in lung cancer [148], hepatocellular carcinoma [149], and prostate cancer [150].

At the signaling level, PDK4 activates the mTORC1-mediated HIF-1α and PKM2 pathways, enhancing the Warburg effect through the cAMP-response element-binding protein/Ras homolog enriched in the brain (CREB/RHEB) cascade [151]. In contrast, RelA/p65, a member of the NF-κB family, suppresses PGC1α-driven PDK4 transcription, thereby increasing PDHC activity and maintaining pyruvate flux into the TCA cycle. This shift abrogates cisplatin resistance via excessive ROS production. However, the transcriptional repressor ZBTB2 inhibits RelA/p65, restoring PDK4 expression through the ZBTB2–RelA/p65–PGC1α axis and ultimately promoting glycolysis, tumorigenicity, and cisplatin resistance [152].

Epigenetic mechanisms also contribute, as hypomethylation of the PDK4 promoter has been reported across multiple cancers, resulting in its upregulation and promotion of tumorigenesis and resistance [153]. Functionally, PDK4 stabilizes KRAS localization at the plasma membrane, activating the MAPK and NRF2 pathways to sustain redox homeostasis and enhance DNA damage repair, thereby reinforcing cisplatin resistance [154, 155].

**Fig. 5:**
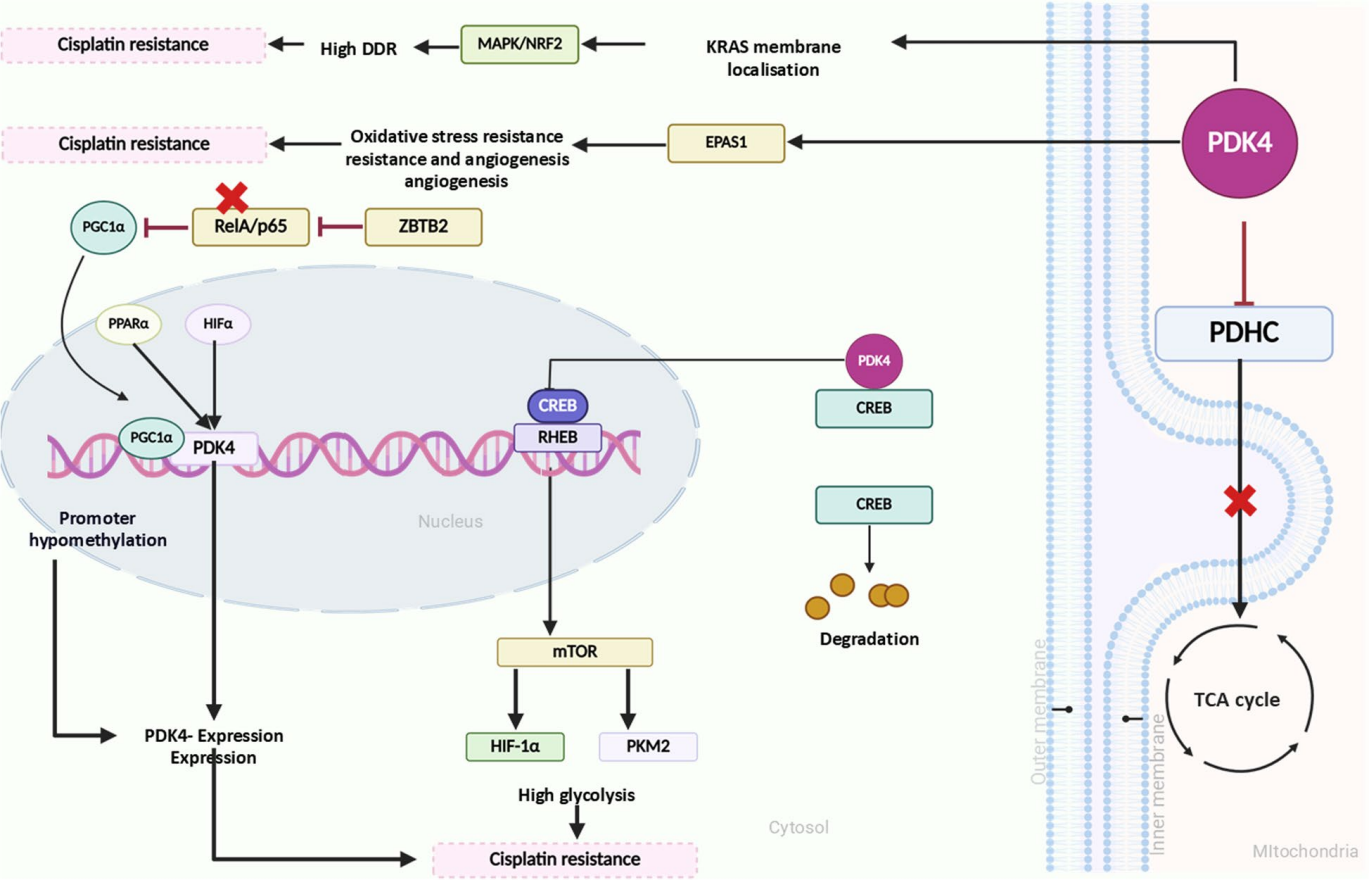
Schematic representation of PDK4 mediated regulation of cisplatin resistance

The functional role of PDK4 in tumorigenesis and cisplatin resistance is paradoxical, with reports of both oncogenic and tumor suppressive effects, as discussed above. These opposing roles are highly context-dependent and are influenced by factors such as the metabolic microenvironment, organ-specific metabolic demands, and cellular states, including epithelial–mesenchymal transition (EMT). For instance, PDK4 overexpression has been reported to promote tumorigenesis in contexts where cancer cells rely on mitochondrial oxidative metabolism, such as in prostate cancer [150]. Conversely, reduced PDK4 expression is associated with increased de novo lipogenesis and tumor progression in hepatocellular carcinoma [149] and lung cancer [156], which is consistent with its regulatory role in fatty acid metabolism. Hence, the role of PDK4 in cancer appears to depend on the tissue of origin and its basal metabolic requirements; cancers arising from tissues with high basal PDK4 expression, where it contributes to metabolic homeostasis, may exhibit tumor-suppressive effects, whereas tumors from tissues with lower basal PDK4 expression may exploit PDK4 upregulation as an oncogenic metabolic adaptation. Furthermore, PDK4 overexpression has been shown to partially prevent TGFβ-induced EMT and associated drug resistance, as metabolic rewiring during EMT is often characterized by increased dependence on oxidative phosphorylation [148]. In addition, the function of PDK4 is linked to oncogenic driver mutations, as studies have reported the role of PDK4 in activating mutant KRAS in KRAS-mutant tumors [156]. Collectively, these findings highlight the multifaceted and context-specific role of PDK4 in tumorigenesis and cisplatin resistance (Fig. 5). Hence, therapeutic targeting of PDK4 must be stratified according to tumor metabolic phenotypes, as indiscriminate modulation may produce divergent outcomes. A comprehensive summary of PDK isoforms and their regulatory factors contributing to cisplatin resistance in major cancers is provided in Table 2.

**Table 2:** Summary of PDK isoforms and its regulatory mechanisms causing cisplatin resistance

PDK Isoform	Main Regulatory Factors	Key Cancers	Resistance mechanisms	Reference
PDK1	FGFR1/BCR-ABL	Lung cancer	High glycolysis	[131]
	HIF-1-α	Burkitt’s lymphoma	High glycolysis	[122]
	Circ ARNT2/miR-155-5p	HCC	High glycolysis	[126]
	ATF3/TGF-β/SMAD	Osteosarcoma	Cancer stemness	[127]
	α5β1 integrin and JNK/IL-8	Ovarian cancer	High glycolysis	[128]
	EGFR	Ovarian cancer	High glycolysis	[34]
	NRF2/ARNT	Melanoma, cervical cancer	Inhibit DNA fragmentation and apoptosis	[155]
	PGC1- α/HSP70/VDAC	Ovarian cancer	High MMP and inhibition of cyt-c release	[133]
	E2F8/PDK1	Lung cancer	High DDR	[134]
PDK2	HK2	Ovarian cancer	High MMP and inhibition of cyt-c release	[136]
	CNNM3	Lung cancer	Cancer stemness	[33]
	p51/p21/E2F/PDK2	Breast cancer	High glycolysis	[136]
	mt-ND mutation/HIF-1- α	HNSCC	High glycolysis	[138]
	TGF-β	HNSCC	Stemness	[139]
PDK3	HIF-1- α/PDK3	Colon cancer	High glycolysis	[141]
	HSF1/PDK3	Gastric cancer	High glycolysis	[142]
	KDM4A/E2F1/PDK3	Prostate cancer	High glycolysis	[143]
	miR-497-5p	Gastric cancer	High glycolysis	[144]
	C14MC	Cervical cancer	High glycolysis	[145]
PDK4	HIF-1- α/PPAR- α/PDK4	Bladder cancer	High glycolysis	[152]
	EPAS1	Lung cancer	Oxidative stress resistance, angiogenesis, high glycolysis	[147]
	CREB/RHEB/mTOR/HIF-1- α	Lung cancer, breast cancer, prostate cancer, pancreatic cancer	high glycolysis	[151]
	ZBTB2/RelA/p65	Glioblastoma	high glycolysis	[152]
	KRAS/MAPK/NRF2	Lung cancer and colorectal cancer cells	Maintain redox homeostasis	[154, 156]

Although PDK isoforms display tissue-specific expression patterns, they share substantial sequence homology (~ 70%), suggesting partial functional redundancy [136]. A study in HNSCC reported that combined silencing of PDK1 and PDK2 resulted in a significant reduction in cancer stemness and increased cisplatin sensitivity, indicating functional redundancy in maintaining the stemness phenotype in cancer cells [139]. In addition, cancer cells often exhibit intratumoral heterogeneity in terms of PDK isoform expression, which may influence the efficacy of isoform-specific inhibitors [157]. Experimental studies suggest the possibility of compensatory regulation among PDK isoforms. In PDK2-knockout mice, in which PDK2 is the predominant skeletal muscle isoform, increased PDK1 expression was observed, indicating an adaptive response [158]. These findings further indicate that the compensatory upregulation of PDK1 was not fully sufficient to restore total PDK activity, yet PDH activity remained lower in these mice than in wild-type mice [158]. Nevertheless, studies directly examining compensatory regulation among PDK isoforms in the context of cancer and therapy resistance remain limited. From a therapeutic perspective, pan-PDK inhibition with dichloroacetate (DCA) may overcome isoform compensation by broadly targeting PDK-mediated metabolic reprogramming in cancer cells, which often rely more strongly on glycolytic metabolism than normal tissues do. However, this broad inhibition may also increase the risk of off-target toxicity in metabolically active normal tissues such as the heart, liver, and skeletal muscle, where PDKs play essential roles in metabolic regulation [120]. Consequently, despite promising preclinical findings and early-phase clinical evaluations, the use of DCA has not progressed to widespread clinical approval, partly because of systemic toxicity, including peripheral neuropathy [159–162]. The choice between pan-PDK inhibition and isoform-specific inhibitors should be guided by the expression profile of PDK isoforms within tumors. Targeting individual PDK isoforms may be more appropriate in cancers with a dominant isoform, whereas pan-PDK inhibition may be preferable in tumors with intratumor heterogeneity in terms of PDK expression.

### Metabolic regulation of PDK

The activity of PDKs is finely regulated by diverse intracellular metabolites, enabling them to orchestrate pyruvate flux into the TCA cycle and thereby influence cisplatin sensitivity. PDK activity is inhibited by allosteric regulation from pyruvate, which typically accumulates because of PDHC inhibition by PDKs. A study in specific skeletal muscle types under high-fat conditions demonstrated a significant increase in PDK4 expression along with a sevenfold increase in pyruvate levels while maintaining approximately 50% PDHC activity. Here, the reduction in PDHC activity is attributed to the reduced efficiency of PDK4 in the presence of elevated pyruvate concentrations [163]. In parallel, pyruvate is also converted to oxaloacetate by pyruvate carboxylase, resulting in the TCA cycle [164]. In addition to pyruvate, other metabolites, such as ADP and NAD⁺, act as inhibitory signals. ADP functions as an allosteric inhibitor of PDK by occupying its ATP binding pocket, thereby preventing PDHC phosphorylation and shifting metabolism toward mitochondrial oxidation. This metabolic rewiring may contribute to the sensitization of cells to cisplatin [165, 166]. Similarly, a high NAD+/NADH ratio demands energy production and suppresses PDK activity by providing a more oxidative environment around PDHC, hindering its phosphorylation by PDK. Conversely, a high NADH/NAD+ ratio favors a reduction in the number of lipoyl groups in the E2 subunit of PDHC, and at the same time, a high acetyl-CoA/CoA ratio causes its acetylation, both of which simultaneously act as allosteric activators of PDK, augmenting its phosphorylating capacity and exerting an inhibitory effect on PDHC [167]. However, the regulatory effects of these metabolites vary across isoforms. NADH and acetyl-CoA together increase PDK1 activity by 200% and PDK2 activity by 300%. NADH alone increased PDK4 activity by 200%, with no further increase upon acetyl-CoA addition. In contrast, PDK3 remains unresponsive to NADH, and its activity is inhibited when NADH is combined with acetyl-CoA [167].

**Fig. 6:**
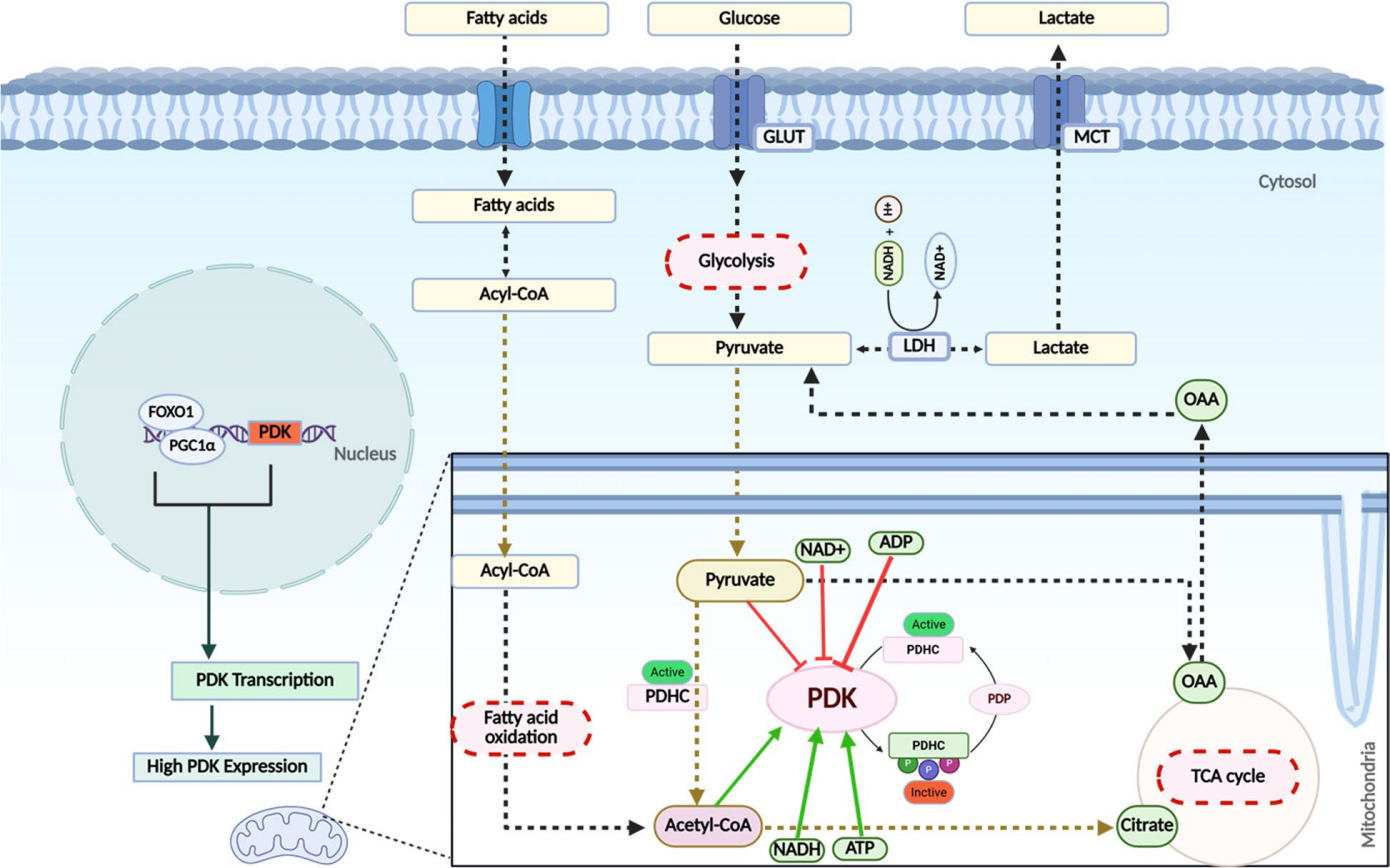
Regulation of PDKs by metabolic intermediates

Collectively, these findings suggest that PDK activity is tightly regulated by a complex interplay of intracellular metabolites and transcriptional mechanisms. Such metabolic reprogramming plays an important role in tumor progression and may influence cellular responses to chemotherapy, including cisplatin sensitivity and resistance. (Fig. 6).

### PDKs and tumor–immune metabolic crosstalk

Metabolic reprogramming plays a critical role in determining therapeutic response by modulating the cytotoxic functions of tumor-associated immune cells through altered immune metabolism and nutrient availability [168]. PDKs, as central regulators of metabolic flux between glycolysis and mitochondrial oxidation, have been increasingly implicated in tumor-immune metabolic crosstalk and the balance between pro- and anti-inflammatory responses [169]. Although studies directly linking PDKs to cisplatin sensitivity through immune modulation are limited, emerging evidence highlights the role of PDKs in immune evasion mechanisms.

In clear cell renal cell carcinoma (ccRCC), elevated expression of PDK2 or PDK3 is associated with increased T-cell infiltration, accompanied by an increased proportion of exhausted CD8⁺ T cells [170]. This may result from tumor-mediated glucose restriction, which limits T-cell substrate availability and suppresses mTOR activity, glycolysis, and IFN-γ production, thereby promoting functional exhaustion while promoting tumor progression [171]. Furthermore, PDK1-driven lactate production has been shown to promote histone H3 lysine 18 lactylation (H3K18la) at the PD-L1 promoter, leading to increased PD-L1 expression and reduced CD8⁺ T-cell infiltration [172]. Lactate accumulation associated with aerobic glycolysis further contributes to an immunosuppressive microenvironment by inducing T-cell exhaustion [173], suppressing interferon-gamma (IFN-γ) secretion by natural killer cells [174], promoting M2-like polarization of tumor-associated macrophages [175], and impairing dendritic cell-mediated immune responses [176]. Similarly, PDK1 overexpression in ovarian cancer suppresses IFN-γ secretion and impairs CD8⁺ T-cell function through dysregulation of the PD-1/PD-L1 axis [177]. Similarly, elevated PDK3 expression in colon cancer has been linked to altered CD8⁺ T-cell infiltration via STAT1 activation and PD-L1 upregulation, whereas combined PDK3 inhibition and PD-1 blockade significantly suppressed tumor growth by restoring CD8⁺ T-cell activity [178].

Beyond immune cells, metabolic reprogramming within cancer-associated fibroblasts (CAFs) and cancer-associated adipocytes (CAAs) further reinforces an acidic, nutrient-competitive tumor milieu. CAFs, in particular, supply glycolytic intermediates to neighboring tumor cells and secrete cytokines, chemokines, and angiogenic factors that collectively support tumor progression [179].

Collectively, these observations suggest that PDK-driven metabolic remodeling not only influences intrinsic tumor cell behavior but also indirectly promotes immune evasion by creating a metabolically restrictive and lactate-enriched microenvironment.

### Inhibitors of PDK

Collectively, all PDK isoforms contribute to cisplatin resistance across diverse cancers, underscoring their importance as potential therapeutic targets. Dichloroacetate (DCA) is the most extensively studied PDK inhibitor, particularly in glycolytic tumors [180]. DCA is a pyruvate analog that binds to an allosteric site within the amino-terminal domain of PDK isoforms, inducing conformational changes in both the nucleotide and lipoyl binding pockets in PDKs [181]. Functionally, DCA synergizes with cisplatin by enhancing glucose oxidation, modulating the mitochondrial membrane potential (MMP), and increasing ROS generation [182, 183]. The fusion of cisplatin with two DCA molecules in axial positions, termed mitaplatin (*cis*, cis-diamminedichlorobis(dichloroacetate)-platinum(IV)), affects both mitochondria and the nucleus in cancer cells in a redox-dependent manner [184]. Other small-molecule inhibitors have also been explored. R-Lipoic acid impairs PDK binding and phosphorylation efficiency toward PDHC [185], whereas its analog CPI-613 disrupts mitochondrial metabolism by targeting PDHC and α-ketoglutarate dehydrogenase/2-oxoglutarate dehydrogenase (OGDH), achieving tumor-selective cytotoxicity [186, 187].

JX06, a synthetic inhibitor, induces cytotoxicity by covalently modifying a hydrophobic pocket adjacent to the ATP binding site of PDK [188]. Another compound, AZD7545, inhibits PDK1 by binding to the lipoyl-binding pocket in its N-terminal domain, thereby preventing its interaction with the E2 subunit of PDHC [189]. A summary of key synthetic inhibitors and their therapeutic potential is provided in Table 3. Despite the development of several novel inhibitors, DCA remains the only PDK inhibitor tested in clinical trials, highlighting the urgent need for further mechanistic studies and translational research into PDK regulation and therapeutic targeting.

**Table 3:** PDK inhibitors in different model systems

Inhibitor	Properties	Model System	References
Dichloroacetate	Pyruvate analog	Solid cancers	[38, 39, 184]
Mitaplatin	DCA fused platinum prodrug	Adenocarcinoma hepatoma	[182]
Lipoic acid	Interaction with lipoyl binding pockets	Prostate cancer, breast cancer, lung cancer	[185]
CPI-613	Interaction with lipoyl binding pockets	Pancreatic cancer, Ovarian cancer, Multiple myeloma, Head and neck carcinoma	[186, 187]
AZD7545	Interaction with lipoyl binding pockets	Diabetic rat hepatocytes Lung cancer	[189]
JX06	Covalent modification of hydrophobic pocket	Lung cancer Neuroblastoma	[188]

### Conclusion and future perspectives

This review provides a comprehensive analysis of the mechanisms underlying cisplatin resistance, with a particular focus on metabolic reprogramming and the emerging role of pyruvate dehydrogenase kinases (PDKs). Although cisplatin remains a cornerstone of cancer therapy, resistance and disease relapse frequently compromise its long-term efficacy. Increasing evidence across multiple cancer types indicates that resistant cells undergo metabolic reprogramming to sustain survival under cisplatin-induced stress. This metabolic plasticity, achieved through the rewiring of central carbon, amino acid, and lipid metabolism, provides important insights into how cancer cells adapt to DNA damage and oxidative stress. PDKs have emerged as critical regulators in this context, functioning as glycolytic switches, modulators of ROS, and maintainers of redox balance, thereby conferring essential adaptive advantages. Collectively, the current evidence highlights the oncogenic potential of PDKs and highlights their role in promoting cancer stemness and therapeutic evasion. Consequently, PDKs represent promising therapeutic targets that could be synergistically combined with conventional anticancer therapies. Although several PDK inhibitors have been reported to have cytotoxic effects in preclinical studies, dichloroacetate (DCA) remains the only compound that has shown both preclinical efficacy and limited clinical evaluation. These findings underscore the need for the development of isoform-selective inhibitors and biomarker-driven strategies to identify patient populations most likely to benefit from PDK-targeted therapies. Furthermore, integrating metabolic interventions, including dietary modulation, with PDK inhibition as part of combination treatment strategies may provide more durable and personalized therapeutic outcomes. However, the clinical application of PDK inhibition must be approached cautiously, as systemic toxicity may arise from off-target effects in vital organs that rely on PDK-mediated metabolic regulation for physiological homeostasis. In summary, this review highlights the pleiotropic roles of PDKs in tumorigenesis and cisplatin resistance, redefining the metabolic paradigm and emphasizing their potential as translationally relevant therapeutic targets.

### Limitations

Our review highlights the role of PDKs in cancer, with particular emphasis on metabolic reprogramming, isoform-specific functions, regulatory mechanisms, and emerging involvement in immune metabolism. However, several limitations should be acknowledged. Despite promising findings from preclinical and early clinical studies, targeting PDK-mediated metabolic reprogramming to increase cisplatin sensitivity presents notable challenges.

First, in non-malignant tissues, PDK isoforms play essential physiological roles, particularly in highly energy-demanding organs such as the heart and skeletal muscle, where they regulate substrate utilization by limiting carbohydrate oxidation and promoting metabolic flexibility [190, 191]. Consequently, systemic PDK inhibition may increase the risk of cardiometabolic toxicity or adverse muscle-related effects. These findings underscore the need for isoform-specific targeting strategies and careful evaluation of potential compensatory mechanisms among PDK isoforms.

Second, although emerging evidence supports a role for PDKs in tumor–immune metabolic crosstalk, studies specifically investigating PDK-mediated immunometabolic regulation in the context of cisplatin therapy remain limited. Therefore, the discussion of PDK-driven immune modulation in this review is necessarily concise and highlights an important area requiring further investigation, particularly in the current era of immunotherapy.

Finally, this review focuses specifically on the role of the PDK axis in metabolic reprogramming. However, tumor metabolic plasticity may enable the activation of compensatory pathways, including fatty acid oxidation and glutamine metabolism, which could attenuate the therapeutic impact of isolated PDK inhibition. Accordingly, the long-term translational implications of selectively targeting PDKs warrant further comprehensive investigation.

## Data Availability

No datasets were generated or analysed during the current study.
